# The interplay between hydrogen bonds and stacking/T-type interactions in molecular cocrystals

**DOI:** 10.1038/s42004-024-01380-3

**Published:** 2024-12-02

**Authors:** Aurora J. Cruz-Cabeza, Peter R. Spackman, Amy V. Hall

**Affiliations:** 1 Department of Chemistry, Durham University, Durham, DH1 3LE UK; 2 School of Molecular and Life Sciences, Curtin University, Perth, WA 6845 Australia

**Keywords:** Crystal engineering, Physical chemistry, Computational chemistry

## Abstract

Supramolecular synthon and hydrogen bond pairing approaches have influenced the understanding of cocrystal formation for decades, but are hydrogen bonds really the dominant interaction in cocrystals? To investigate this, an extensive analysis of 1:1 two-component cocrystals in the Cambridge Structural Database was undertaken, revealing that stacking and T-type interactions are just as, if not more important than hydrogen bonds in molecular cocrystals. A total of 84% of the most common coformers in the dataset are aromatic. When analysing cocrystal dimers, only 20% consist of solely strong hydrogen bonds, with over 50% of contacts involving stacking and T-type interactions. Combining interaction strength and frequency, both hydrogen bond and stacking/T-type interactions contribute equally to the stabilisation of cocrystal lattices. Therefore, we state that crystal engineering and cocrystal design concepts of the future should not solely revolve around supramolecular synthon pairing via hydrogen bonds, but instead consider optimising both hydrogen bonding and stacking/T-type interactions.

## Abstract (web summary)

Hydrogen bond pairing and supramolecular synthon approaches have influenced the understanding of cocrystal formation for decades, but whether or not hydrogen bonds are the dominant interaction in cocrystals has not been extensively studied. Here, the authors perform an extensive analysis of 1:1 two-component cocrystals in the Cambridge Structural Database and reveal that when interaction strength and frequency are combined, hydrogen bonds and stacking/T-type interactions contribute equally to the stabilisation of cocrystal lattices.

## Introduction

**Fig. 1:**
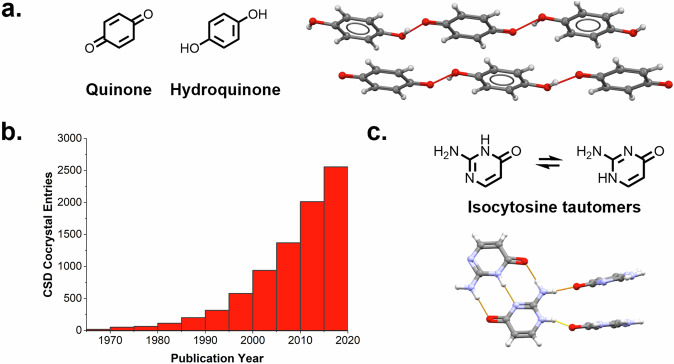
Cocrystals and the CSD. **a** The structure of the first reported cocrystal: quinone and hydroquinone to form quinhydrone (QUIDON)^13,66^. **b** The exponential growth of cocrystal entries in the CSD from 1965 to 2020, plotted from the 8408 dataset of two-component cocrystals (no water, solvents, or zwitterions)^49^. **c** The first cocrystal structure deposited in the CSD (ICYTIN)^22^, consisting of two isocyanate tautomers displaying extensive hydrogen bonding and aromatic stacking.

Cocrystals, generally defined as crystalline solids composed of two or more different neutral molecules in a stoichiometric ratio held together by non-covalent interactions, have applications spanning a wide range of industries, from pharmaceuticals^1–3^ to explosives^4–6^ to optoelectronics^7–9^ to dosimetry^10^. The practice of designing and synthesising molecular compounds is centuries old and so is their isolation in the crystalline state^11,12^. The first ever cocrystal was reported by Wöhler in 1844^13,14^. He produced green crystals containing quinone and hydroquinone from 1:1 solutions which he described as “one of the most beautiful substances known to organic chemistry” (Fig. 1a)^13,14^. Wöhler’s discovery initiated the interest in molecular cocrystals, leading to the report of several cocrystals of phenazone in 1895^15^ and over 300 cocrystals of aromatic compounds in 1922^16^. A year after this, in 1923, the first X-ray structure of an organic molecule was reported which revolutionised what was possible for the characterisation of solid-state materials^17^. From the mid-1920s until the 1950s, Kofler, Kofler, and Kuhnert-Brandstatter synthesised hundreds of multicomponent crystals by contact method thermomicroscopy^14^, with nicotinamide used as a common coformer^18^. In 1965, an important repository to compile the structural data of organic small molecules was born in the form of The Cambridge Structural Database (CSD)^19^. For each entry in the CSD, all crystallographic information is reported, as well as the associated publication. The number of structures deposited in the CSD has grown exponentially since its creation and it now constitutes the single most important source of small molecule crystal data in the world. This is indeed also the case for the number of reported cocrystals in the CSD, shown in Fig. 1b from 1965 to 2020. Whilst cocrystals have been known for centuries, it is perhaps only in the last 20 years or so that their potential to impact materials’ applications has been exploited, with drug delivery as an important example^20,21^. The first cocrystal deposited in the CSD (the year of its creation, 1965) consists of isocytosine tautomers which resemble the base pair interactions in DNA, as shown in Fig. 1c^22,23^.

Common to the quinone:hydroquinone and isocyanate tautomer cocrystals, is the fact that the two distinct molecules forming the cocrystal are held together via strong hydrogen bonds as well as by the strong stacking interactions of the planar molecules. The term ‘intermolecular interactions’ first appeared in the literature in 1954^24^, however, ‘non-covalent interactions’ was only mentioned for the first time in 1974^25^. The main types of non-covalent bonding found in molecular cocrystals are hydrogen bonds (HBs), halogen bonds (HaBs), weak hydrogen bonds (wHBs), and stacking/T-type interactions (STs) involving both aromatic and aliphatic compounds. Below we discuss the use of each of these types of non-covalent interactions in cocrystal design.

The term ‘crystal engineering’ was first coined by Pepinsky in 1955 in a meeting abstract^26^, however, it was initially used in a full scientific paper by Schmidt in 1971^27^. The practice of specifically designing cocrystals based on their potential to form HBs has been commonplace since the popularisation of crystal engineering in the late 1980s and mid-1990s^28,29^. Over the last 30 years, the terms ‘cocrystal’ and ‘hydrogen bond’ appear simultaneously, seemingly due to the strength, directionality, and structural stability that HBs provide, alongside their predictability when considering HB supramolecular synthons and functional group pairings^29,30^. Supramolecular synthons can be either homo (e.g. acid···acid) or hetero (e.g. acid···amide)^31^, with the acid···pyridine and acid···amide heterosynthons being examples of the most robust, illustrated by 1:1 and 2:1 benzoic acid-isonicotinamide cocrystals (CSD refcodes BUDWEC and MOVTOH)^32,33^. Strong HBs and the accumulation of softer wHBs are exceedingly important in cocrystals^34^, however, it is worth considering that they may not be the only significant type of intermolecular interaction that can influence cocrystal design, formation, and stability.

HaBs are specific and directional interactions that demonstrate considerable functionality in organic materials and crystal engineering^35,36^. Halogen bonded homo and heterosynthons are common^29^, however, HaBs have dwindling popularity when compared to HBs, partly as HaBs are restricted by the specific halogen atoms (F, Cl, Br, I) that must be present on the coformer(s). In some cases, HaB cocrystals have shown to be more stable than HB cocrystals^34^, with the example of a 1:1 cocrystal of tetraiodo-1,4-benzoquinone and tetrathiafulvalene, showing enhanced stability because of the I···S HaBs and charge transfer between the stacked components (HUJNUX)^37^. Additionally, when halobenzene molecules stack, their influence is as great as hydrogen bonding^38–40^.

**Fig. 2:**
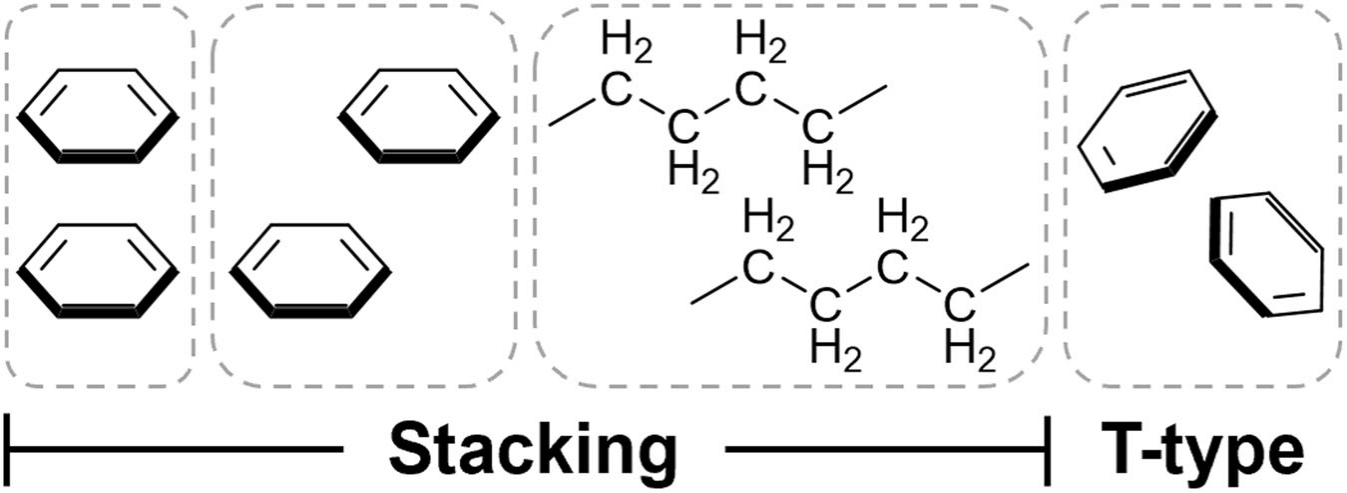
Examples of ST interactions. Left to right: eclipsed face-to-face aromatic stacking, offset face-to-face aromatic stacking, aliphatic alkane stacking, followed by T-type (also known as edge-to-face).

The stacking of compounds can either be aromatic or aliphatic, depending on the nature of the molecules. Aromatic stacking allows aromatic rings to be closer in proximity to optimise intermolecular dispersion^41^, with the most energetically favourable configurations of rings being offset face-to-face and edge-to-face^42^, whereas eclipsed face-to-face stacking is unfavourable due to strong repulsion of the overlapping π electron clouds (Fig. 2)^43^. Aromatic interactions provide the basis of organic optoelectronics in which new semiconductors are synthesised by manipulating the aromatic stacking strengths through pairing of aromatic rings with complementarity, thus resulting in closer stacking distances and improved electrical properties^44^. Cocrystals assembled by aromatic stacking interactions often exhibit intermolecular charge transfer because of the close packing of the π electron donors and acceptors, with perylene and tetracyanoquinodimethane 1:1 and 1:3 cocrystals as an example (PERTCQ01 and TCQPER01)^45^. Furthermore, the role of ST interactions is traditionally ignored in crystal engineering when compared to HBs, which was recently exemplified by Friščić et al. where they concluded that π-systems should be considered as targets for directing cocrystal formation^46^.

As the interest in cocrystal design has increased, crystal engineers have attempted to develop rules to predict and direct cocrystal formation. These rules have undoubtedly been dominated by HBs, with the design of heterosynthons based on them^29,47^. Beyond HBs, HaBs and wHBs have been key players in cocrystal design, however, rules based on controlling dispersion-based interactions (including aromatic and aliphatic stacking and T-interactions) have been consistently underdeveloped, although there are exceptions^48^. In this context, the aim of this study is simple. We seek to quantify the strength of intermolecular interactions in molecular cocrystals and compute their overall contributions to the cocrystal lattice energies. With this, we attempt to answer the question, “Are hydrogen bonds the most important interaction in molecular cocrystals?” by quantitatively analysing the contributions of all interactions to the cocrystal lattice energies.

## Results and discussion

### The cocrystal dataset: overview

We have produced a subset of two-component cocrystals (AB) from the CSD starting from a cocrystal dataset with 9,464 structures – identified in a previous study^49^. The previous study analysed all multicomponent crystals in the CSD and classified them according to the number of components, their protonation state, and their hydration/solvation. Hence, the original dataset only contains two component cocrystals from the CSD with two neutral (and non-zwitterionic) molecular components A and B, where A is larger than B. Structures containing the most common solvents were removed, so the dataset contains mostly cocrystals though less common solvent molecules may also be present. Starting from such a dataset, we took a subset of 3082 structures of cocrystals having 1:1 stoichiometry and Z’ = 1 only, no compounds sitting in special positions, no halogen atoms larger than bromine, and no disorder. This filtering was done to facilitate the computations of interaction energies and their analysis.

### The cocrystal dataset: size distribution

**Fig. 3:**
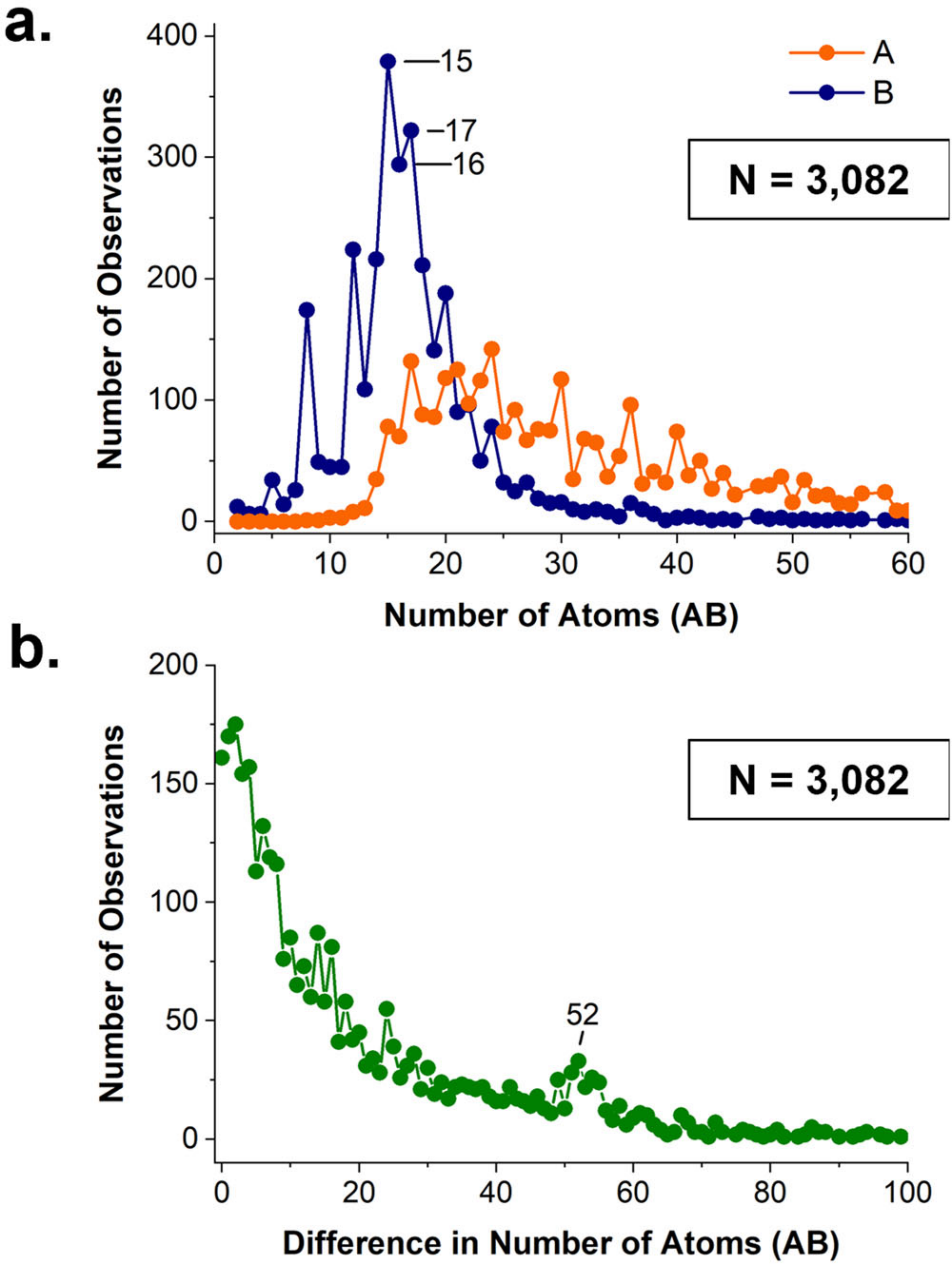
Cocrystal component size. A is the larger component and B is the smaller component in the AB cocrystal. **a** Distributions of sizes for the A and B components in the cocrystals and **b** the distribution of the difference between A and B components.

Here we analyse our dataset of 3082 cocrystals according to the size of its components, A and B. When referring to components within each cocrystal, A is always the larger molecule and B is the smaller molecule. The distribution of sizes of both AB components in our dataset is shown in Fig. 3a. We immediately note that the distribution of sizes for the smaller component B is significantly narrower than that of the larger component A, with peak maxima for B placed at 15, 16, and 17 atoms (Fig. 3a). This may be due to biases of crystal engineers who may often use a limited set of coformers based on structure, availability, and costs for designing cocrystals. The size of both components is smaller than most organic compounds in the CSD. The overall CSD database has a component size distribution peak maximum at 40 atoms and the subset of polymorphs at around 30 atoms^50^; in the cocrystals, we find a peak at about 20 atoms for component B and around 20–30 for component A. This may reflect, again, trends in crystal engineering where cocrystal design has more often been attempted by academic groups working on smaller systems.

The distribution of the difference in atoms between components A and B clearly shows the decay in observations as such difference increases (Fig. 3b). This suggests that cocrystals containing compounds of similar sizes are more common – consistent with the Fabian observation that compounds with complementarity, also in size, are more likely to cocrystallise^51^. There is a small band of observations at around an atom difference of 52 atoms which is related to inclusion structures containing a large host and a small guest.

### The cocrystal dataset: composition

**Fig. 4:**
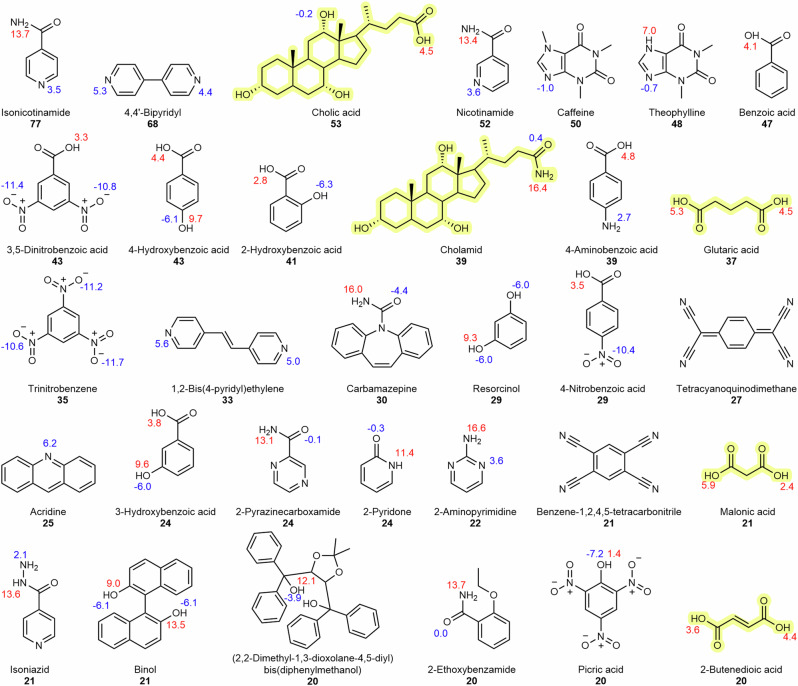
Cocrystal composition. Molecular structures of the top 32 coformers found in the cocrystal dataset in decreasing order of observations (value in bold) along with their common name and p*K*_a_ values (acid values in red, protonated base values in blue). The five aliphatic coformers are highlighted in yellow.

Analysis of the 3082 cocrystal dataset revealed 32 persistent coformers found in at least twenty or more different cocrystal structures (Fig. 4). 84% of the 32 coformers are aromatic. Isonicotinamide is the most common coformer within the dataset as it is found in 77 cocrystals, with its constitutional isomer, nicotinamide, found in 52. Hydroxybenzoic acid constitutional isomers are also found as top coformers with the para isomer having the greatest number of observations (43), followed by the ortho (41), and meta (24) isomers. Only 16% of the 32 coformers were aliphatic. Interestingly, the two most common aliphatic coformers in the dataset are two steroids, namely cholic acid and cholamid, which are well known to form inclusion compounds. Together, these two steroids are found in a total of 92 different cocrystal structures. Following the steroids, dicarboxylic acids are also in this set and include glutaric acid (37 observations), malonic acid (21), and 2-butenedioic acid (20). Analysis by acid/base nature of these top 32 coformers shows that there is an equal number of acids and bases (16 of each). The distribution of p*K*_a_ values (predicted by ChemAxon)^52^ for the acids lies between 1.4 and 18.3 and for the protonated bases is between –11.9 and 6.2, therefore, highlighting the coformers consisting of a range of mild to weak acids and bases.

### The cocrystal dataset: lattice energies

**Fig. 5:**
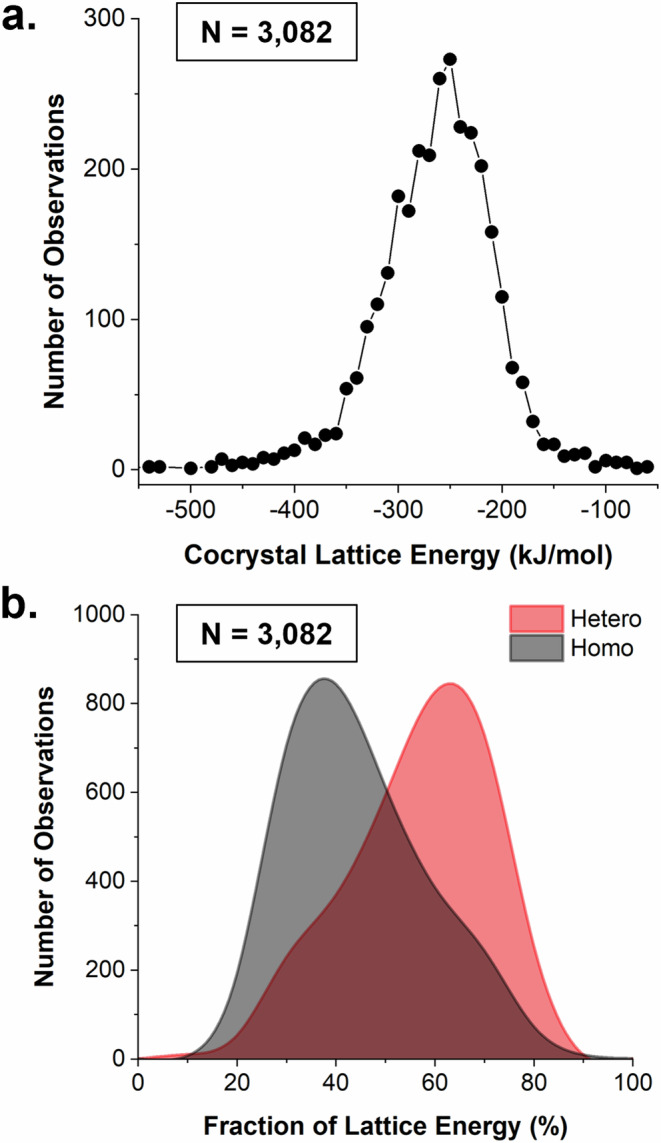
Cocrystal lattice energies. **a** Distribution of cocrystal lattice energies and **b** distribution of hetero- *vs*. homo- fraction of the cocrystal lattice energies.

Lattice energies for the 3082 cocrystal dataset were calculated using the Open Computational Chemistry (OCC) software which offers a command-line implementation of the interaction energy models available in the popular modelling software CrystalExplorer^53^. The lattice energies are computed via summation of dimer interactions until convergence. The computed distribution of cocrystal lattice energies is shown in Fig. 5a with its fraction contributions by homo *versus* heterodimers split in Fig. 5b. There is a maximum in the distribution of cocrystal lattice energies at approximately –250 kJ/mol. Our distribution reflects of course the lattice energies of cocrystals of small molecules, with a relationship between size and lattice energy – similar to trends found in single-component systems^54^. We note that the lattice energies of the cocrystals are given per pair of AB molecules, so this value can be compared to the sum of lattice energies of pure A and B. Lattice energies of pure compounds (similar to these) have been computed to lie around –100 to –110 kJ/mol, so our cocrystal lattice energies are slightly more negative than double that, as expected for cocrystal formation driven by an energy gain^54^.

The partitioning of lattice energies into fractions of homo and heterodimer contributions shows a peak maximum for hetero contributions at around 60% and a peak maximum for homo contributions at around 40%. This suggests that, typically, the heterodimer interactions have an overall greater contribution to the lattice energy than the homodimer interactions (Fig. 5b).

**Fig. 6:**
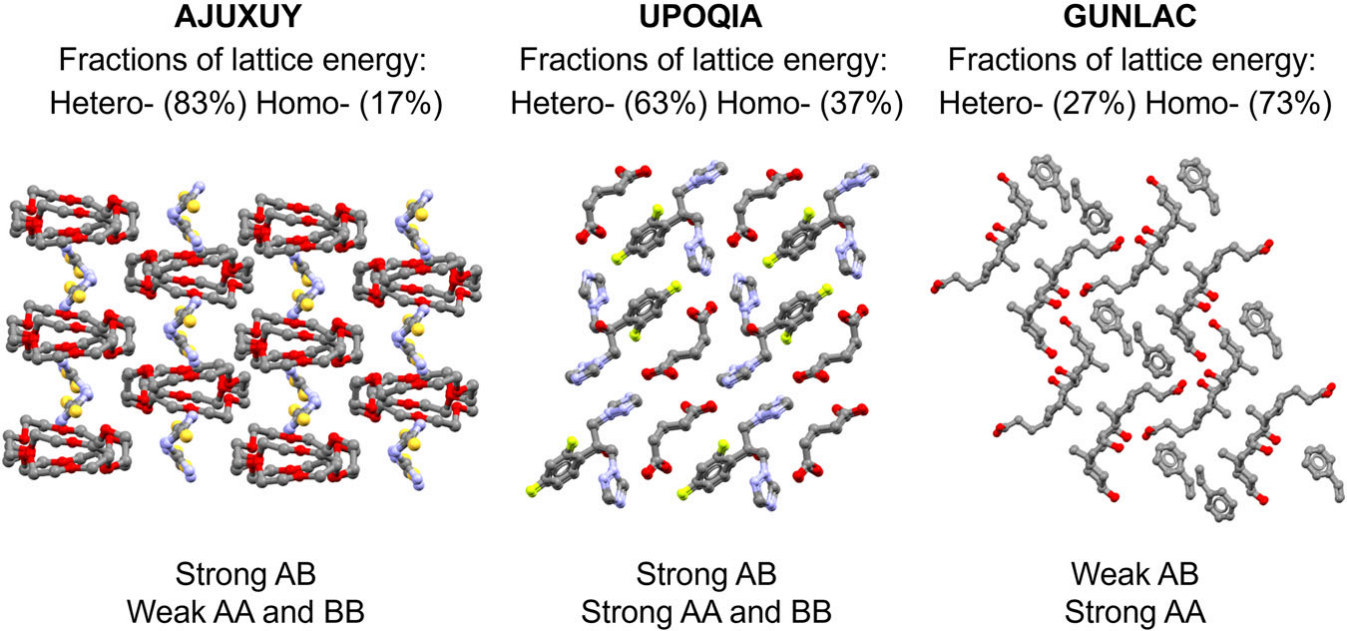
Cocrystal lattice energy examples. Examples of cocrystals with different homo (AA and BB) and hetero (AB) contributions to the lattice energies. Hydrogen atoms are omitted for clarity.

To illustrate how different cocrystals can have markedly different hetero *vs*. homo contributions to the lattice energies, we have selected three illustrative examples from the dataset and shown them in Fig. 6. In the example AJUXUY, the hetero interactions between A and B are very strong while the homo (AA and BB) interactions are overall weak. A and B pack in a highly alternated manner, maximising the AB interactions and their fraction contribution to the lattice energy. In the example UPOQIA, there is generally a balance in the strength of AB, AA, and BB interactions. Here, the AB dimer has a strong contribution of HB energy whilst the AA and BB interactions are based on the stacking of AA and BB through translation symmetry. Finally, in GUNLAC, the homo interactions between the main and larger component A dominate significantly whilst B fills some spaces in the structure. This situation where the homo interactions are much more dominant than the hetero interactions match with inclusion-like structures. Structures of such type tend to have a markedly difference in size between the A and B components with the larger component (and thus the homo interaction) dominating the stabilisation of the lattice and the smaller component playing a minor role.

### Dimers: hierarchy, classification, and examples

**Fig. 7:**
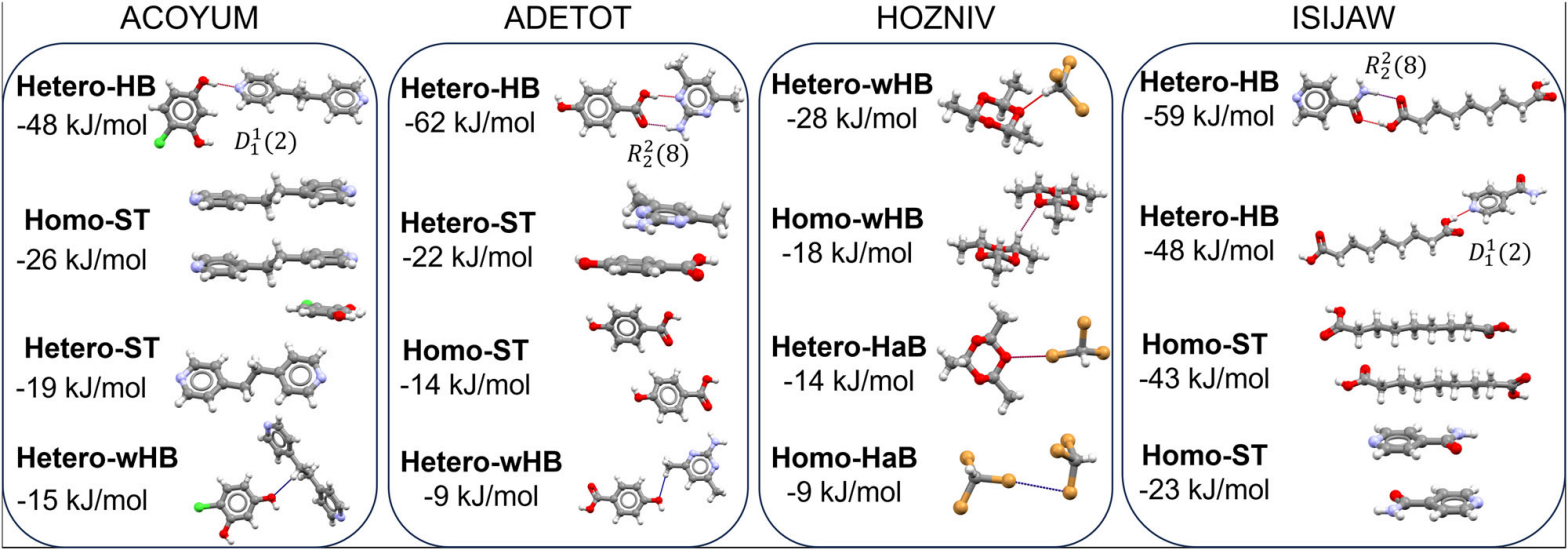
Dimer interaction energy examples. Classification and interaction energies of four dimers in four different cocrystal structures of the dataset.

Almost 1.5 million unique dimer interactions were computed within the 3082 cocrystal dataset with OCC. Of those, 69,089 dimers were found to have interaction energies of less than -1 kJ/mol. In this section, we analyse the structures and interaction energies of the 69,089 dimers. On average, ~22 relevant dimers are analysed per cocrystal structure. Molecule-molecule dimers contain a combination of contacts and thus a ‘pure’ classification of such dimers is not possible – a hierarchy must be established for a meaningful classification. The following hierarchy of interactions was adopted to classify each of the dimers: HB > HaB > ST > wHB. The hierarchy simply assigns priorities for the classification. For example, a dimer containing wHB and HB would be classified as HB since HB is higher in the hierarchy. For a dimer to be classified as ST, at least two ST contacts must exist in the dimer whereas only one contact of the type was required for the other classifications. With this hierarchy, we were able to classify ~40% of the unique 69,089 dimers into these four types (Table 1). The adopted hierarchy correlates well with the average interaction energies for each of the types (Table 1) except for HaB. We note that our dataset excludes the iodine atom from the calculations (no 6-31 G** basis set was available for iodine) causing the average energy of HaB classified dimers to be lower than that expected for HaBs containing iodine. Furthermore, there is only a small number of structures and dimers of molecules containing halogens with close contacts. The contacts with no classification are on average soft interactions of approximately –5 kJ/mol and in most of these interactions, the molecules in the dimers are significantly separated and the repulsion contributions are 0. As clearly seen in Table 1, this classification shows that ST interactions are the most abundant, followed by wHBs and HBs, with HaB being a very small fraction. Further, when symmetry and all dimers (not just the symmetry unique) are taken into consideration, the fractions remain similar. This is simply because the symmetry relations affect all types of dimers equally. To illustrate the effectiveness of our classification rules, we show four classified dimers within the crystal structures of four cocrystals in Fig. 7 together with their interaction energies. Hetero-HB interactions are identified in three cocrystals (ACOYUM, ADETOT, ISIJAW) and are, in all cases, the strongest interactions with stabilisation energies between –48 and –62 kJ/mol. The two ${R}_{2}^{2}\left(8\right)$ motifs are more stabilising than the two ${D}_{1}^{1}\left(2\right)$ motifs. ST interactions are identified in six dimers (both hetero and homo) with stabilisation energies between –14 and –43 kJ/mol. We note that ST interactions are defined broadly and may involve the stacking or T interaction between molecules of any type. The homo-ST in ACOYUM is a classic aromatic stack example with inversion symmetry. The stacking of azelaic acid in the ISIJAW cocrystal is an example of a homo-ST interaction of an aliphatic compound. wHBs are found in three cocrystals (ACOYUM, ADETOT, and HOZNIV) with stabilisation energies ranging between –9 and –28 kJ/mol. Finally, HaB of Br···O and Br···Br are identified in the HOZNIV cocrystal with stabilisation energies ranging from –9 to –14 kJ/mol.

**Table 1:** Classification of 69,089 unique dimers (from 3082 cocrystals) into HB, HaB, ST, wHB and other unclassified interaction types with their average interaction energies

Hierarchy	Dimer Type	All Unique Dimers (69,089)	Fraction all unique dimers	All Dimers (119,396)	Fraction all dimers	Average Interaction Energy (kJ/mol)
1	HB	5623	8.1%	10,481	8.8%	–48 ± 21
2	HaB	290	0.4%	481	0.4%	–14 ± 14
3	ST	14,782	21.4%	25,011	20.9%	–25 ± 17
4	wHB	7312	10.6%	13,763	11.5%	–12 ± 7
–	Unclassified	41,082	59.5%	69,660	58.3%	–5 ± 5

### Dimers: analysis

**Fig. 8:**
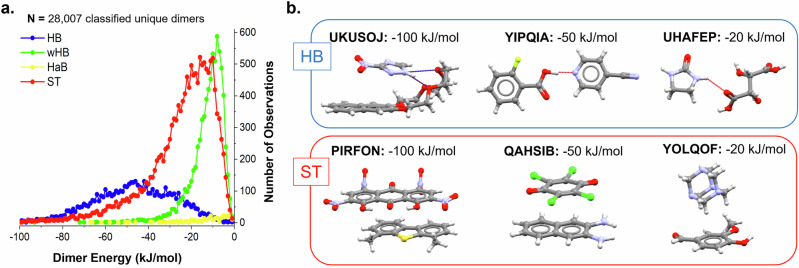
Dimer interaction energies per interaction type with examples. **a** Distribution of dimer interaction energies per interaction type (HBs, wHBs, HaBs and ST). Data on 28,007 classified unique dimers. **b** Examples of hetero HBs and STs of various energies.

In this section, the data on dimers are analysed and meaningful distributions are presented. The distribution of interaction energies by dimer types is shown in Fig. 8a with examples of HB and ST interactions of different strengths in Fig. 8b. Since the HaB interactions are so scarce, they will not be discussed.

We first note the shape of the energy distribution by interaction types (Fig. 8a). The distribution for HBs is broad, for STs narrower and for wHBs the narrowest. A reason for this could be our classification rules. Since the hierarchy of these interactions is HBs > STs > wHBs, dimers classified with interaction types higher in the hierarchy may consist of a blend of interactions. As the classification goes down the hierarchy, the types certainly become more specific – the dimers are less composed of a blend of interactions and are ‘purer’ in nature. This is seen in the example UKUSOJ in Fig. 8b. Whilst the highlighted UKUSOJ dimer is classified as HB, it contains very important ST contributions.

We secondly note the strength of dimer interactions by types (Fig. 8a). The peak maxima for the distributions of the different interaction types (the most common interactions in the dataset) are approximately –50 kJ/mol for HBs, –20 kJ/mol for STs, and –10 kJ for wHBs (Fig. 8a). To shed some light on the interaction types and strengths, examples of HB and ST interactions of high (–100 kJ/mol), medium (–50 kJ/mol) and low (–20 kJ/mol) strengths are shown in Fig. 8b. For the HBs, low-strength HBs are weaker with a suboptimal geometry (UHAFEP), medium-strength HBs are strong with an optimal geometry (YIPQIA) and high-strength HBs are interactions containing strong HBs but also contributions from ST interactions (UKUSOJ). For the STs, we observe a very clear correlation between the ST energy and the size of the molecule. Therefore, the low, medium, and high-strength STs contain, for example, stacking dimers of one, two, and three ring-containing compounds (Fig. 8b). Further to this, aromatic rings with electron-withdrawing groups are found to have strong ST interactions with aromatic rings with electron-donating groups^55^.

**Fig. 9:**
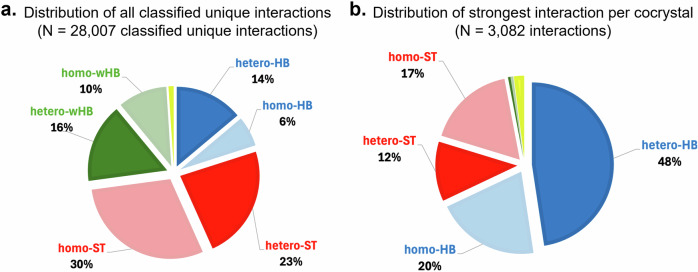
Unique and strongest interactions per cocrystal. **a** Distribution of classified unique interactions (*N* = 28,007) by type. **b** Distribution of strongest interaction per cocrystal (*N* = 3082) by type.

We thirdly note the volume of the interaction types (Figs. 8a and 9a). Whilst STs and wHBs are on average weaker than HB interactions, they are significantly more frequent. This can be seen in Fig. 8a but also is shown more clearly in a pie chart in Fig. 9a. ST interactions account for 53% of all classified unique interactions found in these cocrystals, thus significantly dominating the cocrystals by volume. This is followed by wHBs (26%) and finally HBs (at only 20%). Similar statistics are derived if symmetry is accounted for in the counting of the interactions, which is not shown here for simplicity – our analysis, as described before, only counts symmetry-independent dimers. These hetero-ST motifs are commonly found in cocrystals designed for electronic, optoelectronic, and magnetic applications^56,57^.

The distribution of the strongest interaction per cocrystal by type is shown in Fig. 9b. This pie chart shows that in 48% of the cocrystals, a hetero-HB interaction is the strongest whilst in 20% of the cocrystals, a homo-HB interaction is the strongest. Thus, in 68% of the cocrystals, the HB dimer types are the most stabilising interaction. By contrast, ST interactions are the strongest in 29% of cocrystals, which is significantly less.

**Fig. 10:**
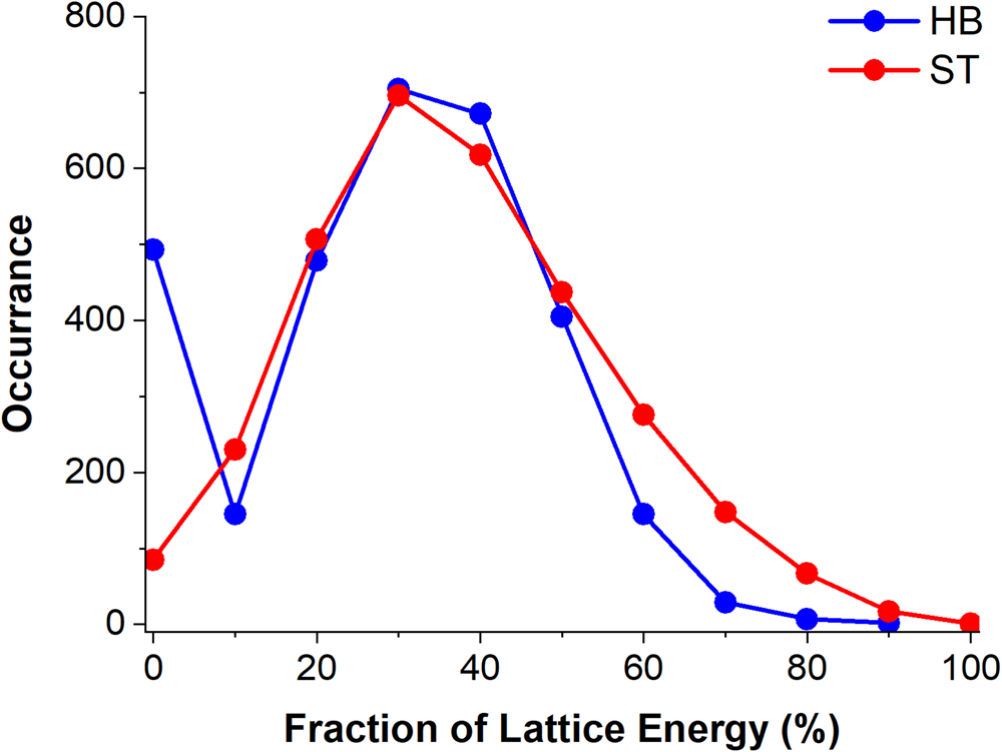
HB and ST interaction contributions to cocrystal lattice energies. Distribution of HB and ST contributions to the lattice energy in the 3082 cocrystal dataset.

To summarise, the analysis of dimer interactions in the 3082 cocrystal structures reveals two clear observations: a) HB interactions are often the strongest interactions and b) ST interactions are often the most common. To analyse these two together, we show the distributions of HB and ST cumulative contributions to the lattice energies of cocrystals in Fig. 10. Remarkably, the distribution of the HB contribution to the cocrystal lattice energy is almost identical to that of the ST contribution (except for values at 0% which are higher for the HB as all compounds will have ST contributions but only those able to HB do). This indicates that taking strength and frequency together, HB interactions are equally as important as ST interactions in molecular cocrystals.

## Conclusions

In response to the question, “Are hydrogen bonds the most important interaction in molecular cocrystals?” the answer is no. Whilst HB interactions in cocrystals are often the strongest, ST interactions are often more common. Taking strength and frequency together, both HB and ST interactions contribute equally to the stabilisation of cocrystal lattices. This is true for current (2023) 1:1 two-component cocrystals in the CSD. We remark, however, that the current cocrystal data are biased towards cocrystals containing relatively small molecules and cocrystals designed using crystal engineering concepts dominated by principles of HB pairing^11^. Even with such a bias, HB interactions still do not dominate the stability of cocrystals. Stronger ST-interactions may indeed lead to cocrystallisation, but this will need to be proven with additional studies from the community. As we develop our understanding of stacking and T-type interactions in molecular crystals and the molecules of the future become larger^46^, the HB may become a secondary role player in cocrystal design. As a community, we must recognise this and consider the incorporation of both HBs and ST interactions in overarching cocrystal design. Such a strategy will more likely lead to more successful observations of novel cocrystals with new properties.

## Methods and computational details

### Dataset

A recent analysis of multicomponent crystals in the CSD revealed 9464 crystal structures of cocrystals containing two main components only (A and B, where the A component is larger than B)^49^. The dataset was further filtered for cocrystal structures with Z’ = 1 and a 1:1 stoichiometry. Crystal structures with components sitting in special positions (two half molecules), containing iodine atoms, or overlapping atoms (due to disorder) were removed. This reduced the number of cocrystals in the dataset from 9464 to 3082 cocrystal structures.

### Dimer and cohesion energy calculations

Dimer calculations were performed on the dataset of 3082 cocrystal structures, all of which completed the process without errors. For this, crystal structures were first retrieved from the CSD and exported as individual CIF files. Dimer energies and cohesive energies for each crystal structure were calculated using the Open Computational Chemistry (OCC) software^58–60^. For each crystal structure, OCC generates all plausible dimers (AB, AA, and BB) within a given shell radius, set to 30 Å in our case. Using the electron densities of the isolated A and B monomers computed using the B3LYP/6-31 G** model^61–63^, OCC then calculates the interaction energies of all unique dimers (energies of dimers related by symmetry are not recomputed) and an overall cohesive (or lattice) energy for the crystal based on a simple summation of all dimer interactions (within a 1 kJ/mol convergence) is computed. This simple method for calculating lattice energies has been shown to perform very well, with computed lattice energies with the OCC method deviating from those computed by benchmark DFT-d methods by only ~6.6 kJ/mol on average^58^. Each unique dimer was classified by its composition as either homo (AA or BB) or hetero (AB). For each cocrystal structure, homo and heterodimer contributions to the cohesive energies were then calculated via independent addition of the relevant dimer interaction energies. Our model predicts lattice energies with good accuracy (<6 kJ/mol) at a very reasonable computational time, splitting the contributions into specific molecule-molecular interactions^64^.

### Data analysis

For data analysis, unique dimers with an interaction energy of less than –1 kJ/mol were considered. This reduced the number of unique dimers in the dataset from almost 1.5 million to 69,089 dimers. The CSD Python API software was used to retrieve structural information from the dimers such as the number of contacts, HBs, graph set notation, SMILES, and the nature of atoms involved in the dimer interaction.

A distance between two atoms was classified as a contact if it was less than the sum of their van der Waals (vdW) radii plus 0.2 Å. Contacts were considered HBs if they involved a hydrogen bond donor (D = O, N, or S), a polarised hydrogen atom and a hydrogen bond acceptor (A = O, N, or S), where the distance between H···A must be less than the sum of their vdW radius and the D-H···A angle larger than 145°. When an HB interaction was found, its graph set was calculated using the method implemented in Mercury^65^. Contacts were classified as HaBs when they involved a halogen donor (F, Cl, or Br) and a halogen bond acceptor (F, Cl, Br, O, N, S) atoms. The remaining contacts were then classified as either wHBs for H···X (where X is an O, N, S, or halogen atom) or STs for X···Y contacts where X and Y could be any heavy atom or hydrogen but excluding H···H contacts. This records CH···C contacts, C···C contacts, and halogen···C contacts typical of stacking or T-interactions.

## Supplementary information

Description of Additional Supplementary Files

Supplementary Data 1

## Data Availability

All data generated or analysed during this study are included in this published article (and its supplementary information file, named Supplementary Data 1).

## Code availability

The software used to compute the dimer data, Open Computational Chemistry, is freely available from Github (peterspackman.github.io/occ/).
