# Supplementary material for: The interplay between hydrogen bonds and stacking/T-type interactions in molecular cocrystals
Source: Commun Chem. 2024 Dec 2;7:284. doi: 10.1038/s42004-024-01380-3 (PMC11612442; doi:10.1038/s42004-024-01380-3)
Supplement: Supplementary file 1 — Description of Additional Supplementary Files [file 42004_2024_1380_MOESM1_ESM.docx]

Description of Additional Supplementary Files

**File name: Supplementary Data 1**

**Description:** This .xlsx file contains all data required to interpret, verify, and extend the research in the article. Included in this data are all 3,082 Cambridge Structural Database refcodes from our cocrystal analysis. Additionally, the supplied data allows the relevant information to reproduce all figures included in the manuscript.
